# SCiPad: evaluating telemedicine via iPad facetime for general spinal cord injury care

**DOI:** 10.1038/s41393-022-00790-1

**Published:** 2022-03-28

**Authors:** Cria-May M. Khong, Elizabeth C. Pasipanodya, Jacqueline Do, Nathan Phan, Daniel L. Solomon, Elyssa Y. Wong, Benjamin Dirlikov, Kazuko Shem

**Affiliations:** 1 Rehabilitation Research Center, Santa Clara Valley Medical Center, San Jose, CA USA; 2 Department of Physical Medicine and Rehabilitation, Santa Clara Valley Medical Center, San Jose, CA USA; 3 T.H. Chan School of Medicine, University of Massachusetts, Worcester, MA USA; 4 School of Medicine, Wayne State University, Detroit, MI USA; 5 Department of Physical Medicine and Rehabilitation, Geisinger Bloomsburg Hospital, Bloomsburg, PA USA; 6 Keck School of Medicine, University of Southern California, Los Angeles, CA USA

**Keywords:** Rehabilitation, Outcomes research

## Abstract

**Study design:**

Uncontrolled clinical pilot study.

**Objectives:**

To assess usage, perceived impact, and satisfaction with a telemedicine program among individuals with spinal cord injury (tele-SCI).

**Setting:**

Community-based.

**Methods:**

Participants (*N* = 83) were recruited from acute SCI inpatient rehabilitation and outpatient SCI care at a community hospital to participate in a 6-month tele-SCI intervention administered by SCI subspecialty board-certified physiatrists via iPad FaceTime. In addition to monthly follow up interview calls, psychosocial and Quality of Life (QoL) measures were collected at baseline and post-intervention. A program satisfaction survey was also collected post-intervention.

**Results:**

Seventy-five percent of participants engaged in tele-SCI visits (Median [*IQR*]: 2.5 [2.0, 4.0]) for a total of 198 tele-SCI visits. Bladder and bowel concerns were the leading topics discussed during tele-SCI visits, followed by neurological, pain, and functional concerns. Tele-SCI users resided further away (Median miles [*IQR*] – 114[73–177] vs. 81[46–116], *p* = 0.023) and reported seeking more clinical advice (Median [*IQR*] – 1.5[0–4.0] vs. 0[0–1.0], *p* = 0.002) compared to non-tele-SCI users. All other clinical utilization, baseline characteristics, psychosocial measures, and QoL did not differ among those who used tele-SCI and those who did not. The satisfaction survey suggested satisfaction with the tele-SCI intervention (89%), study equipment (89%), staff responsiveness (100%), and improved motivation for self-monitoring of health (71%).

**Conclusion:**

Study findings suggest that tele-SCI is a feasible modality for providing general SCI care. Further research is required to examine longer-term efficacy of remotely-provided care among individuals living with SCI.

## Introduction

In the United States, approximately 54 people per day (17,810 annually) sustain a new spinal cord injury (SCI) and an estimated 294,000 persons are living with a SCI [1]. Individuals living with SCI are at higher risk for secondary medical and psychiatric complications, including pain, pressure injuries (PI), and depression, which significantly contribute to poor quality of life (QoL), rehospitalization, and a lifetime of medical expenses [1, 2].

Telemedicine (TM), defined as the exchange of medical information between a clinician and patient from one site to another through the use of technology, addresses some of the barriers to accessing care by facilitating the remote delivery of healthcare services [3, 4]; thus, addressing some of the transportation and economic concerns associated with seeking care [5, 6]. Although individuals with disabilities are generally less likely to use the internet than individuals from the general population, home internet and smartphone use are common, including among individuals with SCI [7, 8]. As such, TM has the potential to improve access to clinical services among both healthy and medically vulnerable populations [9, 10]. Furthermore, in the wake of the COVID-19 pandemic, where TM was widely adopted to reduce the risk of exposure, and with rising rates of home internet and smartphone use, TM is increasingly becoming a more feasible mode of healthcare delivery [11].

Multiple small-scale studies and case reports have found TM to be an efficacious modality for diagnosis, treatment, and monitoring during SCI care [12, 13]. However, most research using TM among individuals with SCI (tele-SCI) has focused on discrete aspects of SCI care (e.g., provision of rehabilitation education [10] and diagnosis or treatment of pressure ulcers and other wounds [14]), rather than on the provision of general SCI care. Thus, extant research on tele-SCI services for individuals with SCI may not be reflective of routine in-person SCI care. As a consequence, there remains a need to evaluate tele-SCI in more ecologically valid conditions and in consideration of the numerous medical concerns encountered by individuals with SCI. Thus, this pilot study was motivated to examine the feasibility, impact, and acceptability of a tele-SCI intervention for the provision of prototypical SCI care. The goals of this project were to 1) describe participants’ usage of tele-SCI and the range of clinical concerns presented during tele-SCI consultations, 2) assess the influence of tele-SCI use on clinical utilization, 3) evaluate the impact of tele-SCI on quality of life outcomes, and 4) assess participants’ program satisfaction. This manuscript provides a follow-up to preliminary findings published using earlier cohorts of the study sample [5, 15] and expands upon those works by reporting findings on a complete study sample and with comparisons of recruited individuals who elected to use tele-SCI against those who did not use tele-SCI. A more expansive sets of analyses on clinical utilization and quality of life measures is also provided.

## Methods

This study received approval from the Institutional Review Board for Human Subjects Protection and all participants provided written informed consent before inclusion in the study.

### Participants

Participants were 83 individuals (65 males, 18 females) with SCI recruited from an acute inpatient rehabilitation unit (ARU) and from an outpatient SCI clinic at a public county hospital in Northern California to participate in a tele-SCI study. Seventy-two participants were recruited from ARU and 11 were recruited from the outpatient clinic. For the participants recruited from ARU, all tele-SCI appointments were completed after discharge from ARU. Study enrollment occurred between January 2015 and August 2018.

Inclusion criteria were: (1) 18 years of age or older, (2) SCI at any neurological level, (3) traumatic or non-traumatic etiology, (4) English-speaking, and (5) discharge to/residing in a private residence within the state of California. Exclusion criteria were inability to provide informed consent and discharge to a medical facility (e.g., skilled nursing facility). Data from part of this cohort were used in previously-published articles [5, 15]; this manuscript reports on data from the full sample of recruited study participants.

### Procedure

At the beginning of the study, each participant received a 9.7-inch Apple iPad, 6-month data plan, hand stylus, and adaptive equipment (e.g., mouth stick and wheelchair mount) if recommended by an occupational therapist. The iPad was selected for its support of end-to-end video and message encryption, and established ease and versatility of use among individuals with physical limitations [15, 16]. All participants were provided with iPads and received training at the start of the study.

Over a 6-month study period, participants had the option of engaging in tele-SCI consultations and visits with a board-certified SCI physiatrist using the video-chat application FaceTime. Participants were also able to contact a research coordinator during business hours who would relay participants medical needs to study physicians. Study physicians responded to non-emergency but urgent requests within 24 hours on weekdays. For medical emergencies, participants were advised to visit a local emergency department (ED) or to call 911.

### Measures

Study assessments were completed over the phone or in-person with research staff. At baseline, demographics and injury related information were recorded. Every month for 6 months, participants completed follow-up interview calls on demographic changes, medical complications, as well as clinical utilization and reasons for in-person and tele-SCI visits. Reasons for tele-SCI visits were divided into the following categories: i) prevention of secondary complications, ii) neurogenic bladder, iii) neurogenic bowel, iv) neurological, v) pain, vi) functional, vii) cardiovascular, viii) psychological, and ix) other.

The following measures were used:

#### Life Satisfaction Index-A (LSIA)

Life satisfaction at baseline and at 6-months was measured using the LSIA, a 20-item survey with facets that include zest for life, fortitude, and congruence between desired and achieved goals [17]. Questions on the LSIA were a 3-point scale ranging from “0” (disagree) to “2” (agree). Negatively-worded items were reversed scored, yielding a range of scores from 0 to 40, with higher scores indicating greater levels of satisfaction. Reliability in this sample was acceptable to good (baseline α = 0.728; follow-up α = 0.806).

#### Patient Health Questionnaire-9 (PHQ-9)

Depressive symptoms were assessed at baseline and at 6-months using the PHQ-9, a validated screening measure for depression [18]. Participants were asked to score each item from “0” (not at all) to “3” (nearly every day). Scores ranged from 0 to 27 with higher total scores indicating greater severity of depression. Reliability in this sample was adequate (baseline α = 0.685; follow-up α = 0.778).

#### Reintegration into Normal Living Index (RNLI)

Global functional status at one month and at study end was assessed using the 11-item RNLI, a survey that evaluates satisfaction in (1) daily and recreational activities, (2) social interactions with family and friends, (3) self-care, and (4) mobility following a debilitating or traumatic injury [19]. Each item of the RNLI is scored from “0” (no reintegration) to “10” (complete reintegration) and items are summed to a total score out of 100. Higher scores indicated better perceived reintegration into daily life. Reliability in this sample was good to excellent (baseline α = 0.886; follow-up α = 0.923).

#### Clinical utilization

Participants’ healthcare-related encounters were assessed monthly over the 6-month study period. In particular, the number of ED visits, hospitalizations, in-office physician visits, tele-SCI encounters, and inquiries seeking clinical advice from any medical professional by phone or email (e.g., urinary tract infections [UTI] advice from primary care physician or daily activities from an occupational or physical therapist) were assessed.

#### Program Satisfaction Survey (PSS)

At study end, participants who engaged in tele-SCI appointments described their experiences and satisfaction with the tele-SCI program in four specific areas: (1) tele-SCI satisfaction, (2) perceived health, (3) equipment satisfaction and, (4) staff satisfaction using a 13-item de novo measure on a 7-point Likert scale from “1” (strongly disagree) to “7” (strongly agree).

### Statistical Analysis

This pilot study’s statistical analyses assessed usage, perceived impact, and satisfaction with a tele-SCI program among individuals with SCI. Descriptive statistics were used to characterize participants, their usage of tele-SCI, and their program satisfaction. Difference tests were used to compare characteristics among study participants who engaged in tele-SCI and those who did not engage in tele-SCI. Difference tests were also used to compare QoL outcomes at multiple study timepoints among individuals who engaged in tele-SCI and those who did not. Continuous normally distributed variables were compared using *t*-tests while continuous non-normally distributed variables were compared using Mann–Whitney *U-*tests. Differences between groups on categorical variables were examined using chi-square tests. For all comparisons, *p*-value of 0.05 was used with no adjustments for multiple comparisons as differences were not examined under a universal null hypothesis [20]. SPSS v24 was used to conduct all statistical analyses [21]. Individuals lost to follow-up were not excluded from analyses.

## Results

Participants were on average 41 years old (*SD* = 16 years). The majority were Caucasian (57%), non-Hispanic (77%), with tetraplegia (70%), and with traumatic etiologies of injury (88%). One participant did not complete any study visits and seven participants were lost to follow-up due to inability to meet study obligations (*n* = 5) and death unrelated to study participation (*n* = 2). At 1-month follow-up, participants were a median of 88.5 [*IQR*- 71.8, 110.0] days from injury (Table 1).

**Table 1:** Sample characteristics by telemedicine usage.

Characteristic	Full cohort (*N* = 83)	Tele-SCI engagers group (*N* = 62)	Tele-SCI non-engagers group (*N* = 21)
Age, mean (SD)	41.59 (16.21)	41.24 (17.08)	41.43 (13.71)
Male, *N* (%)	65 (78.3)	46 (74.2)	19 (90.5)
Race/Ethnicity, *N* (%)			
White	47 (56.6)	39 (62.9)	8 (38.1)
Hispanic	19 (23.0)	11 (17.7)	8 (38.1)
Asian	10 (12.0)	8 (12.9)	2 (9.52)
Black	4 (4.8)	2 (3.2)	2 (9.52)
Native American	1 (1.2)	1 (1.6)	0 (0)
Other	1 (1.2)	1 (1.6)	0 (0)
Unknown	1 (1.2)	0 (0)	1(4.76)
Inpatient recruitment, *N* (%)	72 (86.7)	54 (87.1)	18 (85.7)
Time from Injury at month 1 follow-up (days), median [IQR]	88.5 [71.8, 110.0]	89 [74.8, 109.3]	78.0 [69.0, 120.3]
Traumatic etiology, *N* (%)	73 (88.0)	53 (85.5)	20 (95.2)
Level of injury, *N* (%)			
Cervical	58 (69.9)	43 (69.4)	15 (71.4)
Thoracic	21 (25.3)	15 (24.2)	6 (28.6)
Lumbar	4 (4.8)	4 (6.5)	0 (0)
Complete, *N* (%)	40 (48.2)	27 (43.5)	13 (61.9)
Married, *N* (%)	39 (47.0)	26 (41.9)	13 (61.9)
Higher education, *N* (%)	43 (51.8)	32 (51.6)	11 (52.4)
Employed, *N* (%)	66 (79.52)	48 (77.4)	18 (85.7)
Distance (Miles), median [IQR]	99.0 [60.0, 165.0]	**113.50 [72.75, 176.50]**	**81.0 [45.5, 116.0]**
Discontinued, *N* (%)	8 (9.6)	**2 (3.2)**	**6 (28.6)**
Technology, *N* (%)			
iPad use	21 (25.3)	16 (25.8)	5 (23.8)
Smartphone user	72 (86.8)	54 (87.1)	18 (85.7)
Home internet	75 (90.4)	55 (88.7)	20 (95.2)

Statistically significant characteristics are in bold. IQR is interquartile range.

Sixty-two participants elected to engage in at least one FaceTime tele-SCI visit over the study period. Individuals who chose to engage in tele-SCI visits were more likely to reside significantly further away from the community hospital (Median miles [*IQR*]- 114 [73–177] vs. 81[46–116]; *U* = 434, *p* = 0.023; Hedges *g* = 0.40). Early study termination rates were also significantly higher (Fisher’s exact test: *p* = 0.003, Hedges *g* = 0.91) among those in the tele-SCI non-engagers. Additionally, there was a trend towards more white participants in the tele-SCI engager group (*χ*^2^(1) = 3.242, *p* = 0.072). All other baseline characteristics were not significantly different (Table 1).

Over the course of the study period, participants who utilized the tele-SCI service engaged in a total of 198 tele-SCI visits (Median [*IQR*] = 2.5 [2.0–4.0]) and the median tele-SCI visit duration was 21 min ([*IQR*] = 15–30). Table 2 displays the types and frequencies of medical concerns discussed during FaceTime tele-SCI visits. Overall, the top five medical concerns discussed were bladder (20%), bowel (18%), neurological (14%), pain (11%), and functional (11%) concerns.

**Table 2:** FaceTime TM topics discussed.

Topic area	Frequency	Percent
Bladder	143	20.17%
Bowel	126	17.77%
Neurological	97	13.68%
Pain	81	11.42%
Functional	81	11.42%
Cardiovascular	57	8.04%
Psychological	39	5.50%
Musculoskeletal	27	3.81%
Wound	18	2.54%
Respiratory	11	1.55%
Sleep	8	1.13%
Metabolic	8	1.13%
TBI-related	3	0.42%
Digestive	2	0.28%
Cancer-related	2	0.28%
Reproductive	2	0.28%
Endocrine	2	0.28%
Infection	1	0.14%
Hematological	1	0.14%

Table 3 presents comparisons of clinical utilization across groups. Individuals who used tele-SCI reported seeking more clinical advice over the study period than those who did not use tele-SCI (Median [*IQR*]- 1.5 [0, 4] vs. 0 [0, 1]; *U* = 367.0, *p* < 0.01, Hedges *g* = 0.62)**;** ER visits, physician visits, and hospitalizations were not different between tele-SCI engagers and non-engagers. Table 3 also presents the results of tests of differences in QoL measures by tele-SCI groups. Tele-SCI non-engagers and tele-SCI engagers did not differ at any study time point with respect to the LSIA, RNLI, and PHQ-9. Lastly, Tables 3 and 4 present summaries of participant study satisfaction ratings. All sub-domains of the PSS (Table 3) had median scores greater than 6, suggesting that participants had high rates of satisfaction across measured components of the study. Participants who engaged in tele-SCI had high mean satisfaction scores related to the tele-SCI intervention (89%), the perceived impact of the intervention on their health (71%), study equipment and training (89%), and the study staff’s responsiveness (100%); Table 4.

**Table 3:** Clinical service utilization, quality of life, and program satisfaction by telemedicine groups.

	*N*	Tele-SCI non-engagers group	*N*	Tele-SCI engagers group	Test statistic
Clinical utilization, median [IQR]					
Total ER visits	19	0 [0–1]	62	0 [0–1]	*U* = 478.5, *p* = 0.162
Total physician visits	19	5 [2–7]	62	5 [2.75–9]	*U* = 549.0, *p* = 0.654
Total hospitalizations	19	0 [0–1]	62	0 [0–0]	*U* = 501.0, *p* = 0.201
Total advice visits	19	0 [0–1]	62	1.5 [0–4]	*U* = 367.0, *p* = 0.002
Quality of life measures, median [IQR]					
LSI-A (baseline) *N* = 62	20	25.00 [21.25–32.75]	62	29.00 [24.00–32.00]	*U* = 514.0, *p* = 0.252
LSI-A (6-month) *N* = 20	12	26.50 [18.00–34.50]	57	28.00 [21.00–31.00]	*U* = 317.5, *p* = 0.698
RNLI (1-month) *N* = 60	19	53.00 [64.00–101.00]	60	78.50 [60.50–96.75]	*U* = 538.0, *p* = 0.713
RNLI (6-month) *N* = 19	12	88.50 [77.00–99.25]	57	87.00 [69.50–87.00]	*U* = 283.5, *p* = 0.354
PHQ-9 (baseline) *N* = 61	20	4.00 [1.25–6.75]	61	5.00 [3.00–8.00]	*U* = 499.0, *p* = 0.221
PHQ-9 (6-month) *N* = 20	12	2.5 [0.00–7.50]	57	5.00 [2.00–10.00]	*U* = 246.5, *p* = 0.129
Program satisfaction, median [IQR]					
Tele-SCI satisfaction		–	49	6.40 [5.80–6.80]	N/A
Perceived health		–	49	6.00 [4.00–6.00]	N/A
Equipment satisfaction		–	49	6.40 [6.00–7.00]	N/A
Staff satisfaction		–	49	7.00 [6.50–7.00]	N/A

IQR is interquartile range.

**Table 4:** Program satisfaction ratings among individuals who used tele-SCI.

Domain	Satisfaction statement	Agree	Neither agree or disagree	Disagree	N/A
Tele-SCI satisfaction	A.-The care I received through telemedicine was just as good as seeing my physician or nurse.	87.80%	4.10%	8.20%	0.00%
	B.-I would recommend the telemedicine program	100.00%	0.00%	0.00%	0.00%
	C.-I would like to continue to have telemedicine visits with my physician or nurse	83.70%	4.10%	8.20%	4.10%
	D.-I feel my health has improved because of the telemedicine program.	75.50%	20.40%	4.10%	0.00%
	E.-I was satisfied with the quality of the visual image and audio sound during my telemedicine visit(s).	98.00%	0.00%	2.00%	0.00%
Perceived health	F.-Since receiving the iPad, I have been motivated to monitor my health.	71.40%	16.30%	8.20%	4.10%
Equipment satisfaction	G.-The training I received helped me to understand how to operate my iPad.	75.50%	8.20%	4.10%	12.20%
	H.-The iPad was easy to use.	98.00%	2.00%	0.00%	0.00%
	I.-I am satisfied with my use of the iPad.	98.00%	2.00%	0.00%	0.00%
	J.-The adaptive equipment I received was sufficient for my needs.	85.70%	2.00%	4.10%	8.20%
	K.-The iPad took too much time to use.	2.00%	4.10%	93.90%	0.00%
	L.-I was worried about my privacy with the iPad.	6.10%	8.20%	83.70%	2.00%
Staff satisfaction	M.-Staff responded to my needs sufficiently.	100.00%	0.00%	0.00%	0.00%

Program satisfaction ratings were received from 49 participants.

The mean satisfaction for the domains were 89% for tele-SCI satisfaction, 71% for perceived health, 89% for equipment satisfaction, and 100% staff satisfaction.

Likert scale responses were collapsed across categories to capture general agreement, neutrality, and disagreement with survey statements.

Statements K and L were reverse scored.

Statements A and C do not add to 100% due to rounding.

## Discussion

This tele-SCI study contributes to the literature by examining the feasibility, impact, and acceptability of tele-SCI for the provision of routine outpatient SCI care. As there were no mandated number of tele-SCI visits, the study assessed the characteristics of those who elected to utilize tele-SCI, the concerns they presented during tele-SCI visits, as well as their in-person clinical utilization.

Over the course of this study, nearly 200 Facetime tele-SCI visits were conducted among individuals with SCI and the majority of study participants (75%) chose to engage in at least one FaceTime tele-SCI visit. Participants who used tele-SCI resided on average 40 miles farther from the study site compared to tele-SCI non-engagers, suggesting that distance is an important factor in choosing to use tele-SCI. Similar to that of in-person outpatient care, the study’s remote tele-SCI visits addressed a wide range of concerns, including neurogenic bowel and bladder problems, neurological concerns, and pain [22]. This suggests that tele-SCI may be a suitable treatment modality for addressing a substantial proportion of medical needs.

Individuals who elected to use FaceTime tele-SCI had similar numbers of in-person physician visits compared to those who did not use tele-SCI. Tele-SCI users reported seeking more clinical advice over the study period; thus, tele-SCI users may have used tele-SCI services as adjuncts to in-person care. Despite their higher utilization of non-emergency services, tele-SCI users also had equivalent utilization of emergency care (i.e., ED visits and hospitalizations), suggesting that the increased use of non-emergency care did not translate to fewer acute care needs. Participants who elected to use tele-SCI also did not differ in QoL outcomes; however, tele-SCI users were highly satisfied with the tele-SCI service and reported equivalence with in-person visits for addressing medical concerns. Taken together, participant reports of satisfaction and acceptability with tele-SCI suggests preliminary feasibility of tele-SCI for general SCI care among individuals with SCI, although similar rates of physician visits do not suggest replacement of in-person visits by tele-SCI.

A growing body of literature suggests that tele-SCI can be efficacious for diagnosis, treatment, and follow-up care among individuals with SCI, and may also be a good method for acute and chronic management of SCI, patient and caregiver education, and improved access to healthcare [6, 12]. Although current literature on the effects of tele-SCI for individuals with SCI suggests efficacy for discrete aspects of SCI care [14, 23, 24], the examination of its role in the general SCI care in lieu of the traditional routine in-person care has not previously been established. At a time when tele-SCI was not widely implemented for clinical care, study participants engaged in remote consults for routine and emergent concerns (e.g., pressure sore evaluations, swelling of the lower extremities, last minute prescription refills, bladder and bowel inquiries), were appropriately triaged for in-person care, received medication refills, and were assisted in obtaining durable medical equipment. Since then, the COVID-19 pandemic has prompted a dramatic development and reliance upon tele-SCI services while also imposing new strain on traditional global healthcare systems. In the United States, emergency expansion of the Medicare authorization for tele-SCI during the COVID-19 pandemic facilitated the implementation of various TM approaches in order to minimize the spread of COVID-19, provide alternative access care for routine visits, and promote continuity of care. Our local research study and the broader changes to healthcare that have occurred nationally in response to the COVID-19 pandemic provide evidence for the versatility of tele-SCI and the wide array of services it can accommodate.

### Limitations and future directions

This study has several limitations. First, a significant limitation is that reasons for non-utilization of tele-SCI were not asked as a part of the study design. Thus, factors influencing participants’ decisions to not engage in tele-SCI visits are unknown and may introduce selection bias into the study. Additionally, the reasons for engagers significantly seeking out more clinical advice than the non-engagers are also unknown. Future studies should explore the degree to which individuals already actively seek health-related advice and the types of advice resources they utilize, which may influence decisions to engage in tele-SCI services. Future work may also consider alternative health and wellness outcomes that may be impacted by the ability to readily seek and receive medical advice via tele-SCI.

Future research should also aim to examine barriers to tele-SCI utilization, as these may inform understandings of the adoption of tele-SCI for clinical care. This study was also unable to determine whether individuals who elected to use tele-SCI did so because they had more emergent medical needs during the study, resulting in greater clinical utilization of routine care services. Future study designs should also systematically document new medical concerns over the course of the study period and investigate whether there are differences in the types of medical concerns presented during in-person vs. remote care.

Although this study examined characteristics that differed among tele-SCI engagers and non-engagers, it is not possible to determine whether these pre-study characteristics related to tele-SCI use or to other factors not accounted for by the study design. Tele-SCI use was elective and by self-selection; thus, there is a possibility of selection bias. Additionally, although this study found no association between tele-SCI use and QoL, our findings may not speak to the effectiveness of tele-SCI in impacting QoL outcomes. Future studies may address these concerns through larger sample sizes and randomized control study designs.

As participants who used tele-SCI also sought in-person care, this study was unable to present comparisons in outcomes of tele-SCI vs. in-person care. Future studies examining the impact of tele-SCI on SCI outcomes may consider randomization to exclusively tele-SCI and in-person care groups. Additionally, to examine equivalence or differences in care with treatment modalities, future research may also consider longer follow-up periods. Furthermore, given evidence to suggest underreporting of medical utilization, future studies should corroborate participants self-report of utilization with provider/system records of encounters [25]. Future research should also consider examining differential care needs and the impact of tele-SCI in both the acute and chronic phases of SCI, among individuals with varying levels of experience and familiarity with technology, as well as to explore the influence of demographic factors (e.g., ethnicity, race, and age group). Indeed, in this study, comparisons by race showed a trend towards more white participants in the tele-SCI engager group and, although not significant, there were proportionally more participants who were male (91% vs 74%), Hispanic (38% vs 18%), black (10% vs 5%), and with complete injuries (62% vs 44%) in the tele-SCI non-engagers group versus engager group.

At the time of the study, Android and Apple iOS dominated the US mobile operating system market shares [26]. The provision of a study tablet that used a single operating system and video application may have resulted in decreased adoption compared to using a familiar device that participants already owned. For generalizability of tele-SCI services and easier adoption, permitting multiple operating systems, platforms, and video-capable devices may improve implementation. Finally, as study staff were English-speaking, only English-speaking participants were enrolled. In the US, non-English-speaking communities tend to have less access and receipt of preventative care than English-speaking communities [27]. Thus, having provisions to allow non-English-speaking communities access to tele-SCI studies will further equity in healthcare access and allow for greater generalization of study findings.

## Conclusion

Findings from our pilot tele-SCI study suggest that TM is a feasible modality for providing general SCI care. Tele-SCI engagers were satisfied with the overall tele-SCI services provided and reported equivalence between in-person and remote care. Tele-SCI may offer an effective and efficient approach to addressing a wide range of medical concerns and may aid proactivity in seeking and receiving health care. Additionally, following the COVID-19 pandemic and recent rapid and widespread adoption of TM services, this work lends support to the continued use and expansion of tele-SCI in the post-pandemic period. Further research with larger sample sizes, longer follow-up periods, randomized sampling, and the use of multiple video-capable platforms may provide additional information regarding the efficacy and generalizability of broad-based tele-SCI care among individuals with SCI.

## Data Availability

The datasets generated and/or analyzed during the current study are available from the corresponding author on reasonable request.
